# Rapid shipboard measurement of net-collected marine microplastic polymer types using near-infrared hyperspectral imaging

**DOI:** 10.1007/s00216-023-04634-6

**Published:** 2023-03-16

**Authors:** Aaron J. Beck, Mikael Kaandorp, Thea Hamm, Boie Bogner, Elke Kossel, Mark Lenz, Matthias Haeckel, Eric P. Achterberg

**Affiliations:** 1 GEOMAR Helmholtz Centre for Ocean Research Kiel, Wischhofstr. 1-3, 24148 Kiel, Germany; 2 Institute for Marine and Atmospheric Research Utrecht, Department of Physics, Utrecht University, Utrecht, The Netherlands; 3 Present Address: Agrosphere Institute (IBG-3), Institute of Bio- and Geosciences, Forschungszentrum Jülich GmbH, Jülich, Germany; 4 Present Address: National Park Authority, Virchowstrasse 1, 26382 Wilhelmshaven, Germany

**Keywords:** Atlantic Ocean, Catamaran net, Gyre, Shipboard analysis

## Abstract

Isolation and detection of microplastics (MP) in marine samples is extremely cost- and labor-intensive, limiting the speed and amount of data that can be collected. In the current work, we describe rapid measurement of net-collected MPs (net mesh size 300 µm) using a benchtop near-infrared hyperspectral imaging system during a research expedition to the subtropical North Atlantic gyre. Suspected plastic particles were identified microscopically and mounted on a black adhesive background. Particles were imaged with a Specim FX17 near-infrared linescan camera and a motorized stage. A particle mapping procedure was built on existing edge-finding algorithms and a polymer identification method developed using spectra from virgin polymer reference materials. This preliminary work focused on polyethylene, polypropylene, and polystyrene as they are less dense than seawater and therefore likely to be found floating in the open ocean. A total of 27 net tows sampled 2534 suspected MP particles that were imaged and analyzed at sea. Approximately 77.1% of particles were identified as polyethylene, followed by polypropylene (9.2%). A small fraction of polystyrene was detected only at one station. Approximately 13.6% of particles were either other plastic polymers or were natural materials visually misidentified as plastics. Particle size distributions for PE and PP particles with a length greater than 1 mm followed an approximate power law relationship with abundance. This method allowed at-sea, near real-time identification of MP polymer types and particle dimensions, and shows great promise for rapid field measurements of microplastics in net-collected samples.

**Supplementary Information:**

The online version contains supplementary material available at 10.1007/s00216-023-04634-6.

## Introduction

Millions of metric tons of plastic waste are estimated to enter the ocean annually from coastal sources [23, 32]. Most of the plastic that is produced is less dense than seawater [1], and about 60% of the positively buoyant plastic debris that enters the oceans from land is likely transported offshore by surface currents and winds [31, 36]. Floating plastic debris therefore tends to accumulate within oceanic gyres and restricted coastal waters [31]. During the time the debris remains at sea, large plastics items gradually fragment into smaller pieces —so-called microplastics (MP,plastic particles  <5 mm) — under the combined effects of temperature, UV radiation, and actions by waves and organisms [1, 19, 22].

The ecological effects of microplastics in the ocean are uncertain [2, 5, 56], but thought to be detrimental (e.g., [13, 56] and persistent in the marine environment [27, 37]. The distribution of marine microplastics is highly heterogeneous, and data scarcity can hinder interpretation of transport, fate, and environmental impact [14]. Indeed, Lebreton et al. [30] concluded that there remains a major discrepancy between the amount of buoyant plastic that enters the oceans every year (i.e., millions of tons) and the amount that has been reported at the ocean surface (i.e., hundreds of thousands of tons). Recent analysis using a larger dataset suggested that plastic debris transport to the ocean is in fact orders of magnitude lower than previously estimated [54], and adequate data coverage is essential to constrain the global flux and distribution of plastic debris.

The major bottleneck in workflows for quantifying microplastic particles in marine samples is usually the purification of MPs from natural particles [35] and the analysis of particle polymer type (e.g., [42]. Quantitative analyses of MP polymers by manual Fourier-transform infrared spectroscopy (FTIR) or Raman spectroscopy give high-quality results, but are time-consuming, often limited to a small portion of the sample [38], and may be unable to identify a small portion of the sampled particles due to, e.g., fluorescence [6]. Objective quantitative methods can avoid both false negative and false positive identification commonly associated with visual sorting [25]. Recent advances in particle mapping software help to further reduce operator bias [43, 53].

Hyperspectral imaging (HSI) has emerged in recent years as a promising tool for microplastic identification, with limitations in terms of particle size but substantial advantages regarding cost and speed of analysis [25, 44, 56]. Hyperspectral MP identification may also be possible with reduced or eliminated pre-processing such as organic matter digestion [56]. Similar to short-wave infrared (SWIR, [44], near-infrared (NIR) reflectance spectra of natural materials show broad peaks rather than the sharply defined peaks characteristic of plastic polymers, making it easier to discriminate natural and anthropogenic debris. Hyperspectral imaging has been used successfully to identify polymer types in particles and fibers [12] in soils  [46], marine waters [4, 45, 47] ), fish [56], and beach litter [17, 51].

Hyperspectral imaging of MPs tends to be limited to relatively large particles. Most studies using near-infrared (NIR) or short-wave infrared (SWIR) HSI for MP identification report a size detection limit on the order of 200–300 µm (e.g., [25, 56], although Zhu and colleagues [58] successfully identified particles as small as 100 µm using NIR-HSI. Higher spatial resolution has been reported infrequently [16], and references therein). Nonetheless, most samples of floating MP rely on towed nets with a typical mesh size of  ~300 μm [11, 29] ), so that HSI identification is a technique well-suited for MP identification in such samples. Inexpensive, rapid microplastic identification via NIR-HSI therefore has the potential to greatly expand the global pollution database, for example, through sampling efforts by non-governmental organizations (e.g., [7, 8] and citizen science [21].

The current study focuses on net-collected MPs from the subtropical gyre of the North Atlantic Ocean. Plastic debris in this region was documented for the first time in the 1970s [9], and since then, a number of studies have been carried out in the North Atlantic to understand transport processes and quantify plastic debris (e.g., [10, 15, 28, 29]. Some 20% of the marine inventory of floating plastic debris is accumulated in the North Atlantic [10, 15]. This material appears primarily concentrated in the inner accumulation zone of the North Atlantic subtropical gyre (the North Atlantic “garbage patch”), between the Azores and Bermuda [10]. Plastic inventories within the inner accumulation zone average 400 g km^−2^, with maxima as high as 2500 g km^−2^ [10]. In the same region, Eriksen et al. [15] reported plastic particle abundances of up to 10^6^ km^−2^.

In the current work, we describe a method for rapid NIR-HSI measurement of net-collected MPs at sea in a shipboard laboratory. This approach has the potential to greatly increase the collection of marine MP data and can also provide near real-time data to guide sample collection activities.

## Methods

### Study site

**Fig. 1:**
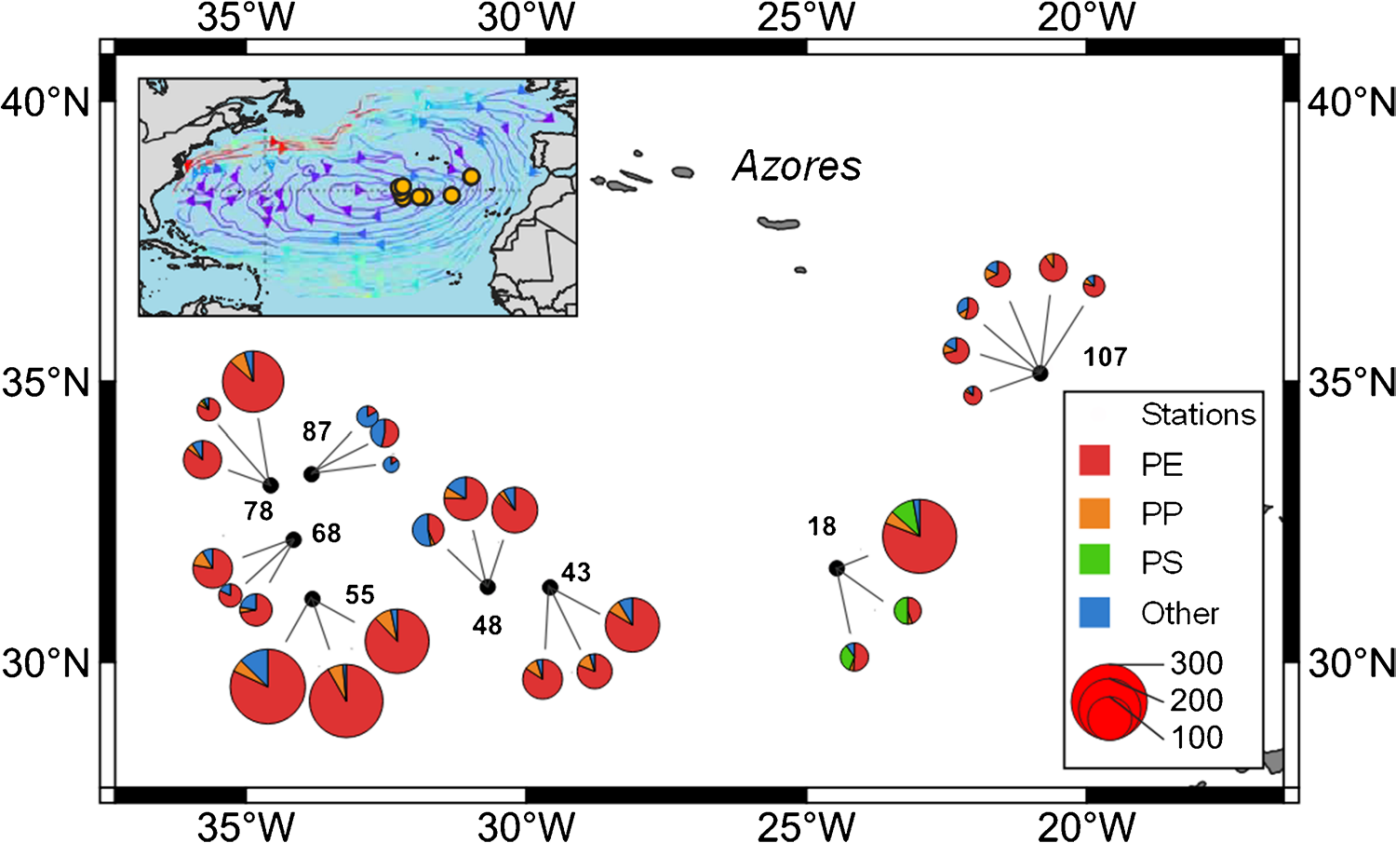
Study site. Filled circles indicate net sampling locations, and numbers are station IDs. Pie charts indicated for each station are the triplicate individual net tows, except station 107, which had six tows (polyethylene, PE; polypropylene, PP; polystyrene, PS). The size of the pie charts indicates the total number of microplastic particles collected in each respective tow. The inset shows the location of the sample locations within the North Atlantic Ocean, with the gyre surface currents (redrawn from [50]. Map drawn with QGIS Version 3.22.13

The current study was conducted during cruise SO279 on R/V SONNE between 04 December 2020 and 05 January 2021. Stations were located within the North Atlantic Gyre south of the Azores, and focused especially on the inner accumulation zone of the gyre (Fig. 1).

### Sample collection and particle mounting

A Neuston Catamaran trawl (Hydro-Bios, Kiel, mouth size 40 × 70 cm) equipped with a mechanical flowmeter and a 300-µm mesh net with a 100-µm cod end was used to collect particles floating at the sea surface. Three net tows were conducted per station at approximately three knots ship speed for 20 min per tow from the side of R/V SONNE. After each tow, the net walls were washed into the cod end with seawater. The cod end was removed, placed in a covered metal pot, and transferred to the lab. Floating *Sargassum* macroalgae was abundant in the study area and was removed from the samples by hand and adhering particles rinsed back into the sample with filtered seawater (FSW; filter cascade with 20, 10, and 1 µm nominal wound-fiber filters; Knaub GmbH, Germany). The sample was then filtered over a 125-µm sieve and rinsed into a glass 500-ml preserving jar with filtered seawater.

Floating plastic particles were picked from the jar by hand or the surface layer of the sample was poured onto a 250-µm sieve with a spoon and left to dry to reduce human bias. Visible particles were picked with forceps and mounted onto adhesive black backgrounds for HSI analysis. The black background reduces reflection in the near-infrared region and is easily removed during the image processing. The test beds were separated into three columns of particles of approximately 1.2 cm wide, slightly less than the camera field of view at the given test bed height.

### Camera system

**Fig. 2:**
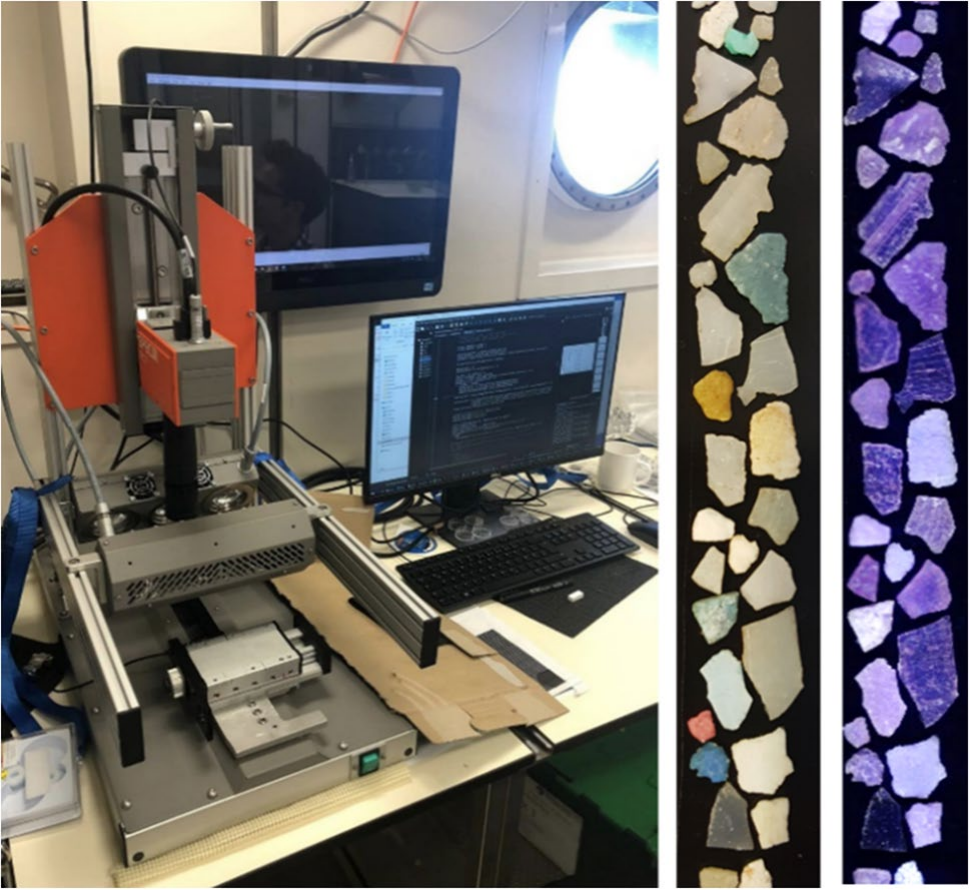
(From left) Benchtop NIR hyperspectral scanning system installed in a shipboard laboratory, and real-color photograph and false-color scan of some large microplastic particles collected in the catamaran trawl. The width of the scan image is about 1 cm

The benchtop NIR-HSI system used in the current study was a Specim FX17 camera (Specim Spectral Imaging Ltd.; Oulu, Finland) mounted on a Specim linear lab bed scanner (Fig. 2). The FX17 linescan camera has a spectral range of 900–1700 nm, with 224 spectral bands and a spatial sampling of 640 pixels. A macro lens was used to achieve a field of view on the order of 1200 µm, giving a pixel dimension of approximately 2–4 µm. Samples were illuminated overhead by two halogen lights at approximately 45° from the front and back of the camera target field. The hyperspectral camera and lab scanner were controlled using Specim’s LUMO software suite. Reference white and dark spectra were measured using a Specim-supplied white reference material and a closed shutter measurement, respectively.

### Image processing

Hyperspectral images were processed using a custom software written in Python. A combination of an edge-finding algorithm (sobol) and a segmentation algorithm (watershed) from scikit-image [49] was used to identify particles in the images. The sobol filter finds the edges of the particles by computing an approximation of the pixel intensity gradients. The detected edges are then used to seed the watershed segmentation algorithm, filling the pixels belonging to each particle. This segmentation method only works with one channel (e.g., black and white images). The mean of the spectrum was therefore taken in each pixel to flatten the hyperspectral image into the appropriate format. This segmentation method worked well as long as the particle edges did not touch.

### Size calibration and measurement

**Fig. 3:**
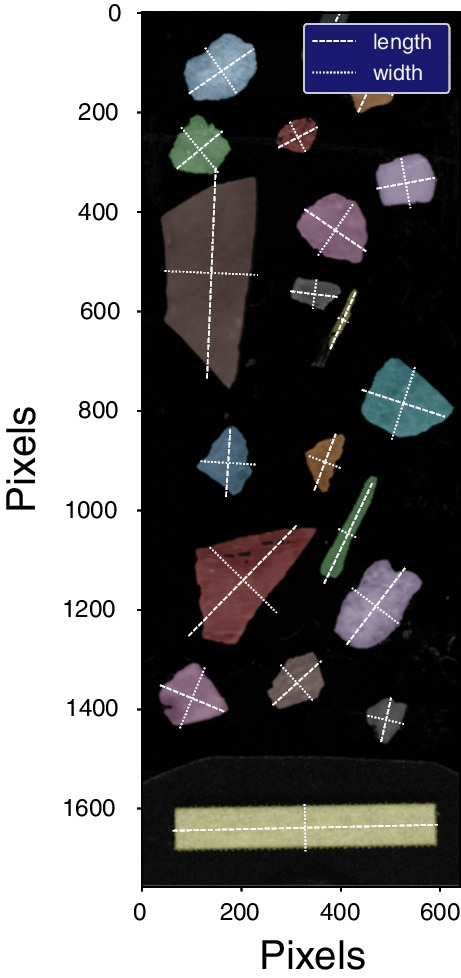
Example scan with particles identified. White dotted lines indicate major and minor axes for particle size determination. The light-colored bar at the image bottom is 10 mm wide

Particle dimensions were calibrated with a 1-cm white reference bar on each scan (Fig. 3). The calibration bar width was used to set the pixel width in the software. Particle length and width were determined by calculating a singular value decomposition on the particle pixels, from which major and minor particle axis lengths were calculated. Other particle properties, such as particle area and perimeter, were calculated using the scikit-image measure class [49]. An example of some scanned particles is shown in Fig. 3, along with the indicated major axes (lengths) and minor axes (widths) of the particles.

### Polymer reference spectra and non-plastic particles

**Fig. 4:**
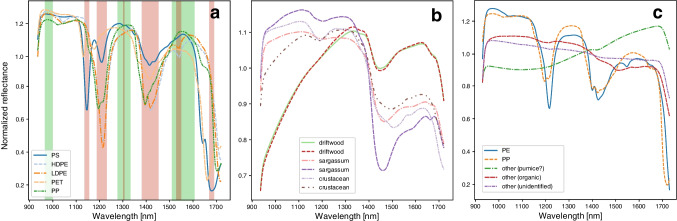
**a** Spectra of reference materials for PS, HDPE/LDPE, and PP. Spectral peak and trough regions are identified in green and red colors, respectively. **b** Spectra for natural net-collected materials. **c** Example spectra for five unknown particles and their putative identification. Spectra are normalized to the average reflectance

A series of virgin plastic polymer beads obtained during the JPI Oceans project BASEMAN [18] were used to establish the reference spectra for polymer identification. The polymers included polystyrene (PS), high- and low- density polyethylene (HD- and LD-PE), and polypropylene (PP) (Fig. 4a). These polymers were chosen for focus because they represent the highest industrial production and abundance in freshwaters and drinking water [26] and are lighter than or close to sea water density: PS (1.04–1.07 g/cm^3^), PE (0.91–0.96 g/cm^3^), and PP (0.90–0.91 g/cm^3^) [3], and therefore most likely to be transported to the central ocean gyre. Because only these three polymer types were included in the reference library, particles identified as “unknown” could be either natural materials or other plastic polymers. The scikit-learn random forest algorithm [39] was used to classify the particle polymer types. The algorithm is trained on the one-hot encoded data from the reference library, taking as features the presence of the peaks and troughs in the spectra.

### Cross-validation of NIR-HSI polymer identification by Raman spectroscopy

A sub-set of 350 particles (~14% of the total particles sampled) taken from NEMICAT tows 14 to 16 were analyzed after the expedition in a land-based laboratory with a Labram HR800 confocal Raman microscope (Horiba Jobin Yvon GmbH, Bensheim, Germany). For each measurement, a photo mosaic of an area with up to 4 cm edge length was recorded, and particles were manually marked on the optical image. An automated multiple point measurement with a 532-nm wavelength laser was then performed at all marked positions. Spectra identification was done with the KnowItAll Horiba edition spectral data base (Bio-Rad, Philadelphia, PA, USA). The identification process was not automated, since fluorescence and noise within a spectrum may cause a poor match with the reference spectrum although a sufficient number of characteristic peaks are present. A fluorescence signal can, for example, originate from dye molecules within a polymer or from organic particles that were mistakenly selected as polymer particles. A high noise level can be observed for strongly weathered polymers or for non-polymeric particles with weak or non-existent Raman modes. Hence, the presence of fluorescence or noise cannot be used to generally identify a particle as polymer or non-polymer. Unidentified particles with high fluorescence were additionally measured with a 785-nm wavelength laser. The fluorescence signal shifts within the spectra depending on the excitation wavelength, and in some cases, a particle identification was possible after the change of the laser wavelength.

## Results and discussion

### Particle spectra

Some example spectra of standard polymers, non-plastics, and unknown particles and their polymer identification by the random forest classifier are presented in Fig. 4. Generally, PE particles showed similar spectra to the reference virgin polymer pellets. Most of these particles showed the peak around 1540 nm, making them easy to identify. Polypropylene particles showed slightly more variation, possibly caused by weathering of the material. Polypropylene particle spectra had a characteristic trough at 1196 nm that was not present for PE. Polypropylene also showed a small trough at 1215 nm, but this was similar to the PE spectra. In some cases, it was difficult to separate the trough at 1215 nm from that at 1196 nm, making it harder to distinguish between the two polymers.

Three spectra classified as “other” materials are shown in Fig. 4c. One particle type was subsequently visually identified as floating pumice stones, which likely originated from the nearby Azores islands (e.g., [20, 48, 55]. The inorganic particle showed a slight increase in reflectance at higher wavelengths, compared with an organic crustacean (shrimp) carapace, which showed a decrease in reflectance above  ~1300 nm. A final example of an unidentified particle is also shown. This particle could not be visually identified, but showed a reflectance spectrum between those of the pumice and shrimp particles. All three spectra were unlike any of the reference plastic particles and were identified correctly as non-polymer by the classification algorithm: the last two spectra were verified by Raman spectroscopy to be non-polymer materials, and the unidentified particle was too fluorescent to identify.

### Polymer classification accuracy

Among the 350 particles analyzed by Raman spectroscopy in a land-based laboratory, 250 were identified as PE, 31 as PP, 16 as non-polymer or unknown spectra and 53 could not be evaluated due to fluorescence or lack of a sufficiently strong signal. The random forest classifier performance was evaluated by using tenfold cross-validation against the particle library positively identified by Raman spectroscopy. The mean probability of detection was calculated (true positive rate) and the mean probability of false alarm (false positive rate). The random forest classifier allows to make a trade-off between these two values. The fraction of decision trees in the random forest voting for a certain polymer, denoted by *P*_*rf*_, is evaluated as a measure of prediction certainty. A threshold value for *P*_*rf*_ can then be selected to achieve a specified true or false positive rate.

**Fig. 5:**
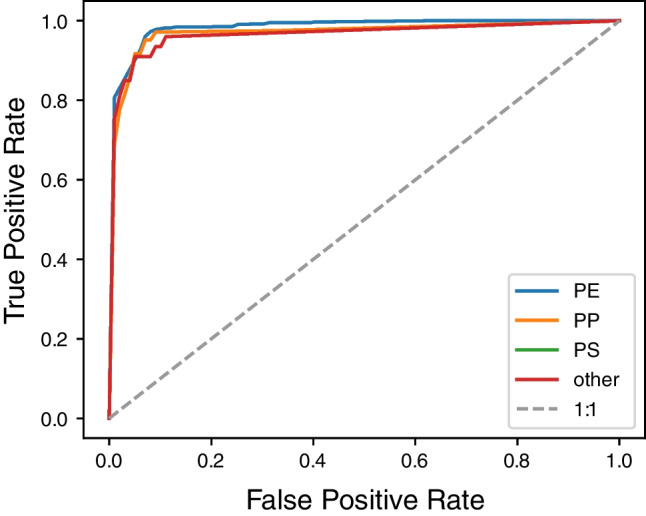
Receiver operating characteristic curve for the identified microplastic polymers

**Fig. 6:**
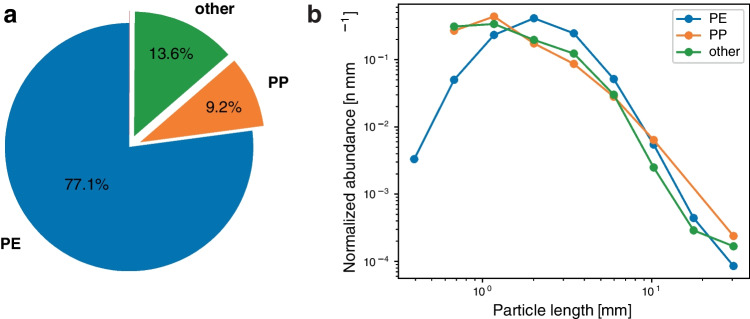
**a** Polymer type proportion of all analyzed particles. **b** Particle size distribution for PE, PP, and the “other” material class, using nine size classes ranging from 0.3 mm (the net mesh size) to 40 mm

The estimated false positive rate is plotted against the estimated true positive rate as calculated from the Raman reference library in Fig. 5. Insufficient data for PS particles was available in the reference library to assess the accuracy of detecting this polymer (i.e., no PS particles were present in the Raman spectroscopy data). Detection of almost all PE particles (e.g.,  >95%), would result in a false positive rate of more than 7% (Fig. 6). A lower detection rate for PE particles (e.g., 92%) would reduce the false positive rate to about 5%. The area under the curves in Fig. 6 is 0.98 for PE, 0.97 for PP, and 0.96 for the other materials, indicating good performance of the classifier. An area of 0.5 (the 1:1 line in Fig. 5) would correspond to an uninformative classifier (in which case randomly guessing whether a particle belongs to a polymer class would be just as good), and an area of 1 would be a perfect classifier. For further analysis, we chose a threshold for *P*_*rf*_ at which the estimated false positive rate remains below 5% (Table 1).

**Table 1:** Detection probability (true positive rate, TPR; false positive rate, FPR) and selection threshold for polymer identification

Material	PE	PP	Others	PE	PP	Others
TPR	0.98	0.86	0.83	0.92	0.92	0.92
FPR	0.07	0.02	0.01	0.05	0.05	0.05
Selection threshold	*P*_*rf, max*_	*P*_*rf, max*_	*P*_*rf, max*_	*P*_*rf*_ > 0.785	*P*_*rf*_ > 0.320	*P*_*rf*_ > 0.243

The spectra for plastic polymers was markedly different from natural materials collected from North Atlantic surface waters such as driftwood, *Sargassum* algae, and crustaceans (shrimp, crabs) (Fig. 4b). Collected microplastic particles showed some evidence of organic biofouling, although this was mostly restricted to crevices (Fig. S1 ) and did not appear to substantially affect the average spectra. Spectral peaks and troughs unique to the different polymers were identified visually (Fig. 4a). The presence of peaks and troughs in the observed particle spectra were identified using the scipy peak finding algorithm [52]. No foam plastic particles were found in the samples. Spectra for high- and low-density polyethylene were indistinguishable and grouped as “polyethylene,” similar to the results of Karlsson et al. [25].

The bulk net samples contained mostly *Sargassum* seaweed and zooplankton, but also up to nearly 300 floating plastic fragments and rope fibers per tow (Table 2). The apparent plastic fragments comprised a range of colors, but were predominantly white (e.g., Fig. 2). False-color scans with the FX-17 camera showed extensive erosion, pits, and cracks of the particles indicative of weathering and fragmentation (e.g., Fig. 2 and Fig. S2). Microplastic particle concentrations ranged between 0.063 and 3.6 particles per cubic meter, excluding stations where the flowmeter was clogged with *Sargassum* (Table 2).

**Table 2:** Summary data of the catamaran net tows, locations, microplastic particles, and microplastic polymers. Additional station data is included in the Supplementary Information

Station	Latitude	Longitude	Volume sampled (m^3^)	Total MP	MP/m^3^	n PE	n PP	n PS	n Other
18–1	31° 39.920′ N	024° 27.287′ W	270	41	0.152	21	2	14	4
18–1	31° 40.476′ N	024° 28.369′ W	196	283	1.444	229	16	30	8
18–1	31° 41.038′ N	024° 29.374′ W	228	38	0.167	17	2	19	0
43–1	31° 19.496′ N	029° 34.347′ W	174	152	0.874	127	12	0	13
43–1	31° 20.462′ N	029° 35.133′ W	24^a^	82	3.417	69	9	0	4
43–1	31° 21.472′ N	029° 35.958′ W	98	63	0.643	51	9	0	3
48–1	31° 19.939′ N	030° 41.100′ W	333	53	0.159	23	2	0	28
48–1	31° 20.671′ N	030° 42.267′ W	325	98	0.302	74	8	0	16
48–1	31° 21.545′ N	030° 43.284′ W	292	108	0.370	95	4	0	9
55–1	31° 07.098′ N	033° 49.016′ W	204	214	1.049	188	19	0	7
55–1	31° 07.953′ N	033° 49.748′ W	176	280	1.591	257	19	0	4
55–1	31° 09.021′ N	033° 50.493′ W	81	293	3.617	240	16	0	37
68–1	32° 10.424′ N	034° 09.152′ W	176	28	0.159	23	0	0	5
68–1	32° 11.403′ N	034° 09.900′ W	244	54	0.221	39	3	0	12
68–1	32° 12.425′ N	034° 10.645′ W	144^a^	81	0.563	63	11	0	7
78–1	33° 08.520′ N	034° 33.673′ W	204	75	0.368	64	4	0	7
78–1	33° 09.608′ N	034° 34.564′ W	269	28	0.104	23	2	1	2
78–1	33° 10.708′ N	034° 35.527′ W	27^a^	194	7.185	168	17	0	9
87–1	33° 20.447′ N	033° 49.999′ W	212	41	0.193	22	0	0	19
87–1	33° 21.646′ N	033° 49.509′ W	206	13	0.063	2	0	0	11
87–1	33° 22.754′ N	033° 49.090′ W	257	24	0.093	4	0	0	20
107–1	35° 08.367′ N	020° 49.280′ W	297	24	0.081	13	3	0	8
107–1	35° 09.270′ N	020° 48.266′ W	333	34	0.102	23	5	0	6
107–1	35° 10.310′ N	020° 47.104′ W	291	40	0.137	36	4	0	0
107–1	35° 11.257′ N	020° 46.044′ W	285	35	0.123	25	4	0	6
107–1	35° 12.198′ N	020° 44.991′ W	308	24	0.078	19	2	0	3
107–1	35° 13.206′ N	020° 43.862′ W	272	18	0.066	15	1	0	2

^a^Net clogged with Sargassum weed, flowmeter blocked

#### Polymer distribution and particle sizes

Microplastic particle concentrations were highest south of the Azores and lowest at the station farthest east (Fig. 1), consistent with accumulation within the gyre interior. However, marked variability was also evident, with replicate tows at individual stations collecting particle numbers that varied by up to a factor of seven (e.g., tows 1-3 at Station 18-1; Table 2). Microplastic polymer types for each tow are shown in Table 2, and the overall proportion is shown in Fig. 6a. The majority of particles were identified as PE (77.1%), with a smaller proportion classified as PP (9.2%). Some particles could not be assigned to a certain polymer and were therefore classified as the ‘other material’ category (13.6%). There was no obvious spatial trend in polymer types (Fig. 1), except for PS, which was only detected at the first station sampled.

A histogram of particle size distribution, as normalized abundance versus length, is plotted for PE, PP, and the “other” material class in Fig. 6b. The amount of particles *n* in each bin is normalized to the bin width, to obtain the particle size distribution in terms of a normalized abundance in *n/*mm (e.g., [10].

For larger particle lengths (~2 mm and larger), the particle size distributions for PE and PP seem to follow approximately a power law relationship, as noted in previous studies [24]. This means that the particle abundance scales with $\propto {l}^{-\alpha }$, where $l$ is the particle length and $\alpha$ is the power law exponent. We calculate $\alpha =3.41\pm 0.08$ for PE, and $\alpha =2.80\pm 0.22$ for PP. As a reference, Cozar et al. [10] calculated $2.93\pm 0.08$ for the global ocean. Local estimates of the particle size distribution shape and slope for different polymers can provide important information on degradation and fragmentation processes [24]. The hyperspectral imaging system allows for rapid quantification of this information and can provide valuable information for future microplastic studies.

Lower abundance of PE and PP particles smaller than 1–2 mm was observed. This has been reported in multiple earlier studies (e.g., [10, 40], and possible causes are related to vertical mixing in the water column of smaller particles [41], removal of smaller particles due to biofouling/ingestion [34], smaller particles escaping the net mesh [33], or sampling bias due to smaller particles not being observed during sample preparation.

## Conclusions

Near-infrared hyperspectral imaging shows very good performance for rapid analysis of large microplastics (i.e.,  >300 µm, and up to 12 mm in the narrowest dimension). The NIR-HSI system is inexpensive relative to most FTIR and Raman spectroscopy devices (~50 k€ versus 500 k€). Sample analysis is fast relative to these other techniques, making it possible to analyze hundreds to thousands of particles in a day. Together, this suggests that NIR-HSI has the potential to relieve bottlenecks in the analytical workload of global microplastic studies. Since the technique works well for large particles, it is particularly well-suited for net-collected particles and those collected during citizen science activities.

The system is light and portable enough that it can be transported to field laboratories, such as research ships, and the microplastic particles can be analyzed soon after collection. Mounting and imaging the collected particles is also simple, reducing the need for technical expertise. The automated particle identification and polymer analysis procedures reduce operator input and improve objectivity. However, the visual particle selection from bulk net samples still introduces a source of user error, and future method developments should focus on eliminating user bias during the particle selection process, for example, by performing a complete digestion of non-plastic material and imaging the entire residual material.

The results of the current study were focused on a very limited set of polymer types. While this is appropriate for offshore regions where transport is limited by particle polymer buoyancy, application in other environments such as beaches will require extension of the polymer library to include more dense polymers as well. Previous reports suggest that the NIR signatures for many additional polymers are sufficiently unique to allow successful discrimination among types (e.g., [51].

A couple changes to the current identification procedure may improve the overall workflow in the future. First, the image segmentation algorithm can possibly be made more accurate by considering the full spectral image, as opposed to segmenting a black and white image as was done here as a preliminary step. This might help detect some of the translucent particles which are hard to detect on the black and white images and would reduce merging errors for overlapping particles. This will be important if manual particle picking and mounting is eliminated in favor of complete digestion and full residue imaging.

Second, the classification algorithm can be improved in the future by including more reference data. Since the reference data in this study was limited, the classification algorithm presented here only uses the presence of prescribed peaks and troughs to keep the number of features low. With enough training data, the entire spectrum could be fed to a classification algorithm instead, with the algorithm determining internally which patterns in the spectrum are most important to distinguish between different materials.

Field-collected samples contain both smaller particles (although perhaps not net samples) and aged or weathered MPs. In addition to a classification library that includes a more diverse range of polymers, it is not yet clear what effect surface weathering has on NIR spectra. The results of the current work suggest that weathering effects do not have a major impact, at least on the key spectra features used for polymer identification on the large MPs analyzed here. However, these spectra features decrease in strength as particle size decreases, and it may be challenging to identify polymer types by NIR-HSI in smaller particles. Weathering effects may also become more important as the spectra quality decreases with particle size.

Finally, MP identification by NIR-HSI may have some advantages compared with other polymer identification techniques, in addition to being low cost and high throughput. For example in the current work, some 15% of the cross-checked particles were unidentifiable by Raman spectroscopy, largely because of fluorescence. Such fluorescence can originate from plastic additives like dyes, and a Raman non-identified particle can still be a polymer and may be correctly identifiable by NIR-HSI.

## Supplementary Information

Below is the link to the electronic supplementary material.

Supplementary file1 (DOCX 1709 KB)
