# Supplementary material for: Rapid shipboard measurement of net-collected marine microplastic polymer types using near-infrared hyperspectral imaging
Source: Anal Bioanal Chem. 2023 Mar 16;415(15):2989–98. doi: 10.1007/s00216-023-04634-6 (PMC10285013; doi:10.1007/s00216-023-04634-6)
Supplement: Supplementary file 1 — Supplementary file1 (DOCX 1709 KB) [file 216_2023_4634_MOESM1_ESM.docx]

**Supporting electronic information**

Rapid shipboard measurement of net-collected marine microplastic polymer types using near-infrared hyperspectral imaging

Aaron J. Beck^1^*, Mikael Kaandorp^2,3^, Thea Hamm^1^, Boie Bogner^1^, Elke Kossel^1^, Mark Lenz^1^, Matthias Haeckel^1^, Eric P. Achterberg^1^

1. GEOMAR Helmholtz Centre for Ocean Research Kiel, Wischhofstr. 1-3, 24148 Kiel (Germany)
2. Institute for Marine and Atmospheric Research Utrecht, Department of Physics, Utrecht University, Utrecht (The Netherlands)
3. Current address: Agrosphere Institute (IBG-3), Institute of Bio- and Geosciences, Forschungszentrum Jülich GmbH, Jülich (Germany)

* Corresponding author: ajbeck@geomar.de, +49 4316001289

Contents:

2 Figures

1 Table


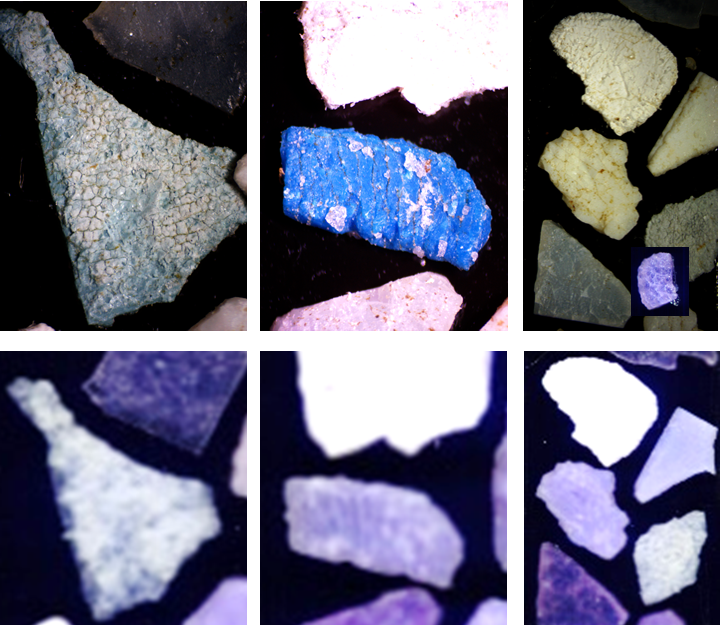


Figure S1. Paired light microscope (upper) and false-color NIR (lower) images of select particles. Brown biofouling material is visible in the cracks on some of the particles.


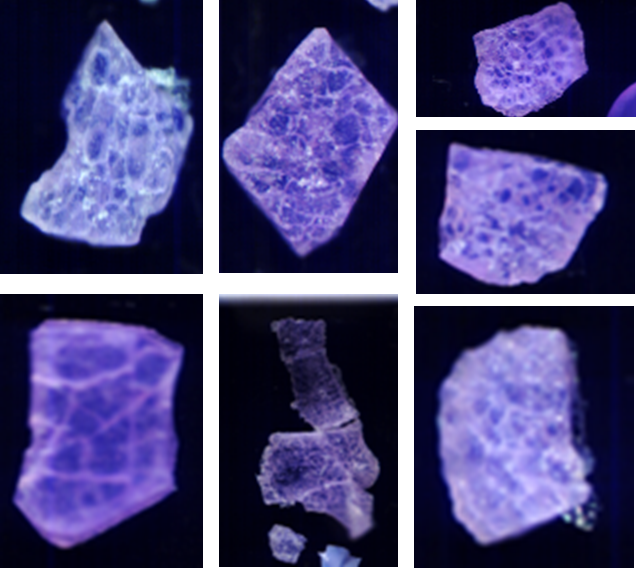


Figure S2. False-color NIR images of example particles showing cracks and fragmentation.

Table S1. Summary data of the catamaran net tows, locations, microplastic particles, and microplastic polymers.

| **Station** | **Net_tow_ID** | **Date / Time UTC** | **Depth (m)** | **Latitude** | **Longitude** | **Wind Dir** | **Wind Speed (m/s)** | **Volume sampled (m^3^)** | **Total MP** | **MP/m^3^** | **n PE** | **n PP** | **n PS** | **n Other** |
| --- | --- | --- | --- | --- | --- | --- | --- | --- | --- | --- | --- | --- | --- | --- |
| 18-1 | NEMICAT-01 | 13/12/2020 13:19 | 5502 | 31° 39.920' N | 024° 27.287' W | 286.4 | 5.8 | 270 | 41 | 0.152 | 21 | 2 | 14 | 4 |
| 18-1 | NEMICAT-02 | 13/12/2020 13:46 | 5503 | 31° 40.476' N | 024° 28.369' W | 304.3 | 5.6 | 196 | 283 | 1.444 | 229 | 16 | 30 | 8 |
| 18-1 | NEMICAT-03 | 13/12/2020 14:11 | 5503 | 31° 41.038' N | 024° 29.374' W | 312.6 | 5.5 | 228 | 38 | 0.167 | 17 | 2 | 19 | 0 |
| 43-1 | NEMICAT-04 | 17/12/2020 07:08 | 4264 | 31° 19.496' N | 029° 34.347' W | 229.7 | 5.4 | 174 | 152 | 0.874 | 127 | 12 | 0 | 13 |
| 43-1 | NEMICAT-05 | 17/12/2020 07:36 | 4254 | 31° 20.462' N | 029° 35.133' W | 223.2 | 5.7 | 24^a^ | 82 | 3.417 | 69 | 9 | 0 | 4 |
| 43-1 | NEMICAT-06 | 17/12/2020 08:11 | 4261 | 31° 21.472' N | 029° 35.958' W | 224.8 | 5 | 98 | 63 | 0.643 | 51 | 9 | 0 | 3 |
| 48-1 | NEMICAT-07 | 17/12/2020 22:00 | 4279 | 31° 19.939' N | 030° 41.100' W | 282 | 6.9 | 333 | 53 | 0.159 | 23 | 2 | 0 | 28 |
| 48-1 | NEMICAT-08 | 17/12/2020 22:31 | 4144 | 31° 20.671' N | 030° 42.267' W | 254 | 6.5 | 325 | 98 | 0.302 | 74 | 8 | 0 | 16 |
| 48-1 | NEMICAT-09 | 17/12/2020 23:02 | 3917 | 31° 21.545' N | 030° 43.284' W | 255.2 | 6.7 | 292 | 108 | 0.370 | 95 | 4 | 0 | 9 |
| 55-1 | NEMICAT-10 | 18/12/2020 19:08 | 4422 | 31° 07.098' N | 033° 49.016' W | 215.7 | 6.5 | 204 | 214 | 1.049 | 188 | 19 | 0 | 7 |
| 55-1 | NEMICAT-11 | 18/12/2020 19:33 | 4426 | 31° 07.953' N | 033° 49.748' W | 210.4 | 7.4 | 176 | 280 | 1.591 | 257 | 19 | 0 | 4 |
| 55-1 | NEMICAT-12 | 18/12/2020 20:01 | 4404 | 31° 09.021' N | 033° 50.493' W | 210.3 | 5.9 | 81 | 293 | 3.617 | 240 | 16 | 0 | 37 |
| 68-1 | NEMICAT-13 | 19/12/2020 23:10 | 4121 | 32° 10.424' N | 034° 09.152' W | 195.2 | 8.7 | 176 | 28 | 0.159 | 23 | 0 | 0 | 5 |
| 68-1 | NEMICAT-14 | 19/12/2020 23:39 | 4120 | 32° 11.403' N | 034° 09.900' W | 174.8 | 7.8 | 244 | 54 | 0.221 | 39 | 3 | 0 | 12 |
| 68-1 | NEMICAT-15 | 20/12/2020 00:09 | 4099 | 32° 12.425' N | 034° 10.645' W | 191.8 | 7.8 | 144^a^ | 81 | 0.563 | 63 | 11 | 0 | 7 |
| 78-1 | NEMICAT-16 | 21/12/2020 09:20 | 3413 | 33° 08.520' N | 034° 33.673' W | 340.4 | 10.5 | 204 | 75 | 0.368 | 64 | 4 | 0 | 7 |
| 78-1 | NEMICAT-17 | 21/12/2020 09:51 | 3132 | 33° 09.608' N | 034° 34.564' W | 343.8 | 12.2 | 269 | 28 | 0.104 | 23 | 2 | 1 | 2 |
| 78-1 | NEMICAT-18 | 21/12/2020 10:24 | 3208 | 33° 10.708' N | 034° 35.527' W | 354.4 | 10.3 | 27^a^ | 194 | 7.185 | 168 | 17 | 0 | 9 |
| 87-1 | NEMICAT-19 | 22/12/2020 09:48 | 3486 | 33° 20.447' N | 033° 49.999' W | 11.3 | 10.8 | 212 | 41 | 0.193 | 22 | 0 | 0 | 19 |
| 87-1 | NEMICAT-20 | 22/12/2020 10:16 | 3397 | 33° 21.646' N | 033° 49.509' W | 15 | 10.9 | 206 | 13 | 0.063 | 2 | 0 | 0 | 11 |
| 87-1 | NEMICAT-21 | 22/12/2020 10:43 | 3380 | 33° 22.754' N | 033° 49.090' W | 358.9 | 10.6 | 257 | 24 | 0.093 | 4 | 0 | 0 | 20 |
| 107-1 | NEMICAT-22 | 28/12/2020 06:18 | 5245 | 35° 08.367' N | 020° 49.280' W | 6.9 | 8.6 | 297 | 24 | 0.081 | 13 | 3 | 0 | 8 |
| 107-1 | NEMICAT-23 | 28/12/2020 06:47 | 5256 | 35° 09.270' N | 020° 48.266' W | 3.1 | 5.6 | 333 | 34 | 0.102 | 23 | 5 | 0 | 6 |
| 107-1 | NEMICAT-24 | 28/12/2020 07:18 | 5253 | 35° 10.310' N | 020° 47.104' W | 8.2 | 5.7 | 291 | 40 | 0.137 | 36 | 4 | 0 | 0 |
| 107-1 | NEMICAT-25 | 28/12/2020 07:47 | 5249 | 35° 11.257' N | 020° 46.044' W | 25.4 | 6.1 | 285 | 35 | 0.123 | 25 | 4 | 0 | 6 |
| 107-1 | NEMICAT-26 | 28/12/2020 08:16 | 5250 | 35° 12.198' N | 020° 44.991' W | 11.8 | 7.8 | 308 | 24 | 0.078 | 19 | 2 | 0 | 3 |
| 107-1 | NEMICAT-27 | 28/12/2020 08:46 | 5255 | 35° 13.206' N | 020° 43.862' W | 350.6 | 10.5 | 272 | 18 | 0.066 | 15 | 1 | 0 | 2 |
| ^a^ Net clogged with sargassum weed, flowmeter blocked | | | | | | | | | | | | | | |
